# Novel Genetic Variants in *PATL*2 Corresponding to Different Clinical Phenotypes of Female Infertility

**DOI:** 10.7150/ijms.109085

**Published:** 2025-06-23

**Authors:** Xiaotao Yang, Xiangrui Shi, Jing Wang, Jingying Guo, Yinhu Huang, Pan Tang, Yu Zhao, Yanxi Li, Wei Liu, Qinghua Zhang

**Affiliations:** 1 Reproductive Medicine Center, Daping Hospital, Army Medical University, Chongqing, 400042, China.; 2 Institute of Immunology, Army Medical University, Chongqing, 400038, China.

**Keywords:** primary infertility, oocyte maturation arrest, *PATL*2 variants, molecular dynamics simulation, structural damage

## Abstract

PATL2, an RNA-binding protein and a translational repressor, plays a crucial role in maintaining mRNA homeostasis during female gametogenesis and early development of embryos. Rare pathogenic variants of its encoding gene have been implicated as causative factors for oocyte, zygote, and embryo maturation arrest (OZEMA), which results in female primary infertility and failed IVF or ICSI attempts. In this study, we identified multiple *PATL*2 variants carried by three patients from two unrelated families: compound heterozygous missense variants comprising novel c.1373T>C (p.I458T), and reported c.877G>T (p.D293Y); unprecedented homozygous missense variants of recurrent c.839G>A (p.R280Q). Molecular dynamics simulations revealed that variants I458T and D293Y severely damaged structural integrity of the PATL2 protein, strongly suggesting a more pronounced functional impairment than the other variant, R280Q. These computational results are in a good consistency with the corresponding clinical phenotypes and offer a plausible explanation for previously observed decrease of protein abundancy associated with the reported variants in *PATL*2. Our findings provide more insights into the significant impacts of both novel and recurrent *PATL*2 variants on female infertility and failed assisted reproduction.

## Introduction

Successful human reproduction relies on (i) mature oocytes and sperms, (ii) positive fertilization, and (iii) normal embryonic development. Any deficiencies in these processes would lead to infertility, which is clinically defined as pregnancy failure after at least one year of regular sexual life without any contraceptive measures. The global incidence rate of infertility ranges from 12.5% to 15%, with more than 50% cases attributed to female infertility 1. Fortunately, the advent of assisted reproductive technologies (ART), such as in vitro fertilization (IVF) and intracytoplasmic sperm injection (ICSI), have revolutionized the treatment of infertility, enabling numerous couples with primary or secondary infertility to conceive. A subset of female patients, however, have undergone recurrent IVF or ICSI failures due to oocyte meiosis deficiency (OMD), a form of primary infertility characterized by the production of immature oocytes 2-4.

Human oocyte production is a lengthy and intricate process that progresses through the germinal vesicle (GV), meiosis I (MI), and meiosis II (MII) stages. In each menstrual cycle, the GV oocytes in antral follicles are sensitive to luteinizing hormone (LH) simulation, which triggers the resumption of meiosis, followed by germinal vesicle breakdown (GVBD) in the MI stage and extrusion of the first polar body (PB1) in the MII stage 1, 4. Only MII oocytes are regarded as mature oocytes ready for sperm binding, with meiosis being arrested again until fertilization occurs. Human oocyte maturation arrest leading to female infertility may occur at any a stage form GV to MII, which was first described in 1990 5, but the genetic causes for it have only been gradually uncovered in recent years.

*TUBB*8 was the first identified human gene being associated with this disorder, with its pathogenic variants accounting for 30% of individuals with MI arrest in a study performed in 2016 6, 7. Shortly afterwards, variants in *ZP*1 or *ZP*3 were found responsible for empty follicle syndrome 8, 9, while *PANX*1 variants were linked to oocyte death 10, 11. Since 2017, autosomal recessive inherited homozygous or compound heterozygous variants in *PATL*2 have been reported to be associated with a spectrum of phenotypes including oocyte maturation arrest, fertilization incompetence, failed zygotic cleavage, and halted early embryonic development 12-17, underscoring the critical role of this protein in oogenesis and embryogenesis. More recently, a comprehensive evaluation and systematic review of 229 genes implicated in non-syndromic female infertility was published, providing a valuable guide for the genetic diagnosis of reproductive disorders 18.

PATL2, a highly expressed protein in oocytes and early embryos, is involved in the regulation of mRNA translation and stability. Studies utilizing model organisms, such as mice and *Xenopus*, have demonstrated that the absence or dysfunction of PATL2 leads to defects in oocyte development, including the formation of smaller or abnormally structured eggs that fail to progress through critical maturation stages. In mice, *Patl2* knockout leads to oocyte maturation arrest, characterized by morphological and developmental abnormalities in both oocytes and zygotes, ultimately resulting in female infertility. In *Xenopus*, overexpression of the PATL2 homolog P100 induces translational repression and blocks oocyte maturation at the GV stage. These findings reveal the highly conserved role of PATL2 across species and its essential function in oocyte maturation 14, 19. Moreover, PATL2 may interact with several proteins, such as CPEB1, DDX6, and EIF4E, which are typical RNA-binding proteins involved in the maintenance of oocyte mRNA stability in *Xenopus* and mice 14, 19, 20, suggesting that PATL2 is part of a complex network responsible for regulating mRNA homeostasis during oogenesis. Even with these reported data, the precise mechanisms by which PATL2 regulates oocyte meiosis progression and the functional consequences of the identified deleterious variants remain poorly understood.

In this study, we identified two *PATL*2 variants harbored by three patients from two unrelated families, including compound heterozygous variants comprising a novel missense variant, c.1373T>C (p.I458T), and a previously reported missense variant, c.877G>T (p.D293Y), as well as a homozygous missense variant of c.839G>A (p.R280Q), which has been documented but its homozygosity has not. To assess the potential impact of these variants on the structural integrity and stability of the PATL2 protein, we conducted molecular dynamics (MD) simulations. Our analyses revealed that these variants significantly compromised its structural stability compared to the wild-type protein, suggesting a potential molecular mechanism underlying the observed clinical phenotypes, although further functional validation is required to confirm pathogenicity.

## Materials and Methods

### Human subjects and ethical approval

Three individuals from two unrelated families diagnosed with primary infertility were recruited from the Reproductive Medicine Center in Daping Hospital. All patients were provided a written informed consent. This study was approved by the reproductive medicine ethics committee of Army Medical University.

### Sequencing and bioinformatics analysis

Peripheral blood for genomic DNA isolation was collected from the affected individuals and other three members (the parents and another sister) in family 1 as well. Exon library construction, whole exome sequencing (WES), bioinformatics analysis and Sanger sequencing of the exons in question were carried out by Annoroad Gene Technology Co., Ltd. (Beijing, China).

### Analysis of sequence variants

Allele frequences of the detected variants were searched in 1000 Genomes (www.internationalgenome.org), Exom Aggregation Consortium (ExAC) browser (exac.broadinstitute.org), and Genome Aggregation Database (gnomAD, version 3.1) Browser (gnomad.broadinstitute.org). Pathogenicity of corresponding variants were predicted using Sorting Intolerant From Tolerant (SIFT, sift.bii.a-star.edu.sg), Polymorphism Phenotyping (Polyphen-2, genetics.bwh.harvard.edu/pph2/), and Mutation Taster (www.mutationtaster.org). Possible impacts to protein structural stability were predicted using the webserver of DUET 21.

### Evaluation of oocyte and embryo phenotypes

Oocytes were obtained from the affected individuals and a control patient diagnosed with tubal infertility. Morphology of the oocytes, their fertilization status, oocyte cleavage patterns, and subsequent embryonic development were meticulously assessed under a ZEISS Axio vert. A1 inverted microscope. The GV, MI, and MII oocytes correspond to the oocytes with an intact germinal vesicle, without GV or polar body, and with an extruded polar body, respectively. Only MII oocytes were considered mature and suitable for IVF or ICSI treatments.

### Molecular modeling

A full-length 3D model of PATL2 was downloaded from Alphafold Protein Structure Database (AlphaFoldDB) 22, followed by discarding the highly mobile N-terminal domain (residues 1-289). The 3D models of the D293Y and I458T mutants were obtained by simple residue replacement using *PyMOL* (v2.4.0) 23. We did not build a model for the p.R280Q variant because the affected residue locates in a region with low predictive confidence according to *AlphaFold*2 24, which would otherwise generate an unreliable structure.

### MD simulations

The C-terminal domain (residues 290-543) of wild-type PATL2 and the mutants of D293Y and I458T were subjected to MD simulations using *Gromacs* (version 2018.8) 25. After hydrogen addition using *pdb2gmx*, the protein molecule was explicitly solvated with TIP3P water molecules in a periodic cubic box with 10 Å away from the protein edges. The CHARMM36 force field 26 were applied for the constructed system, which was neutralized by adding appropriate amount of Na^+^ or Cl^-^ ions. To mimic the physiological saline milieu, the solution concentration of NaCl was adjusted to 0.15 M. After energy minimization, the system was slowly heated to 300K in an NVT ensemble, followed by separated 2-ns equilibrations in both NVT and NPT ensembles. Finally, production simulations were conducted for 200 ns in the NPT ensemble with trajectory snapshots recorded every 20 ps. Cluster analysis was performed using *Cluster*, a plugin in the package of *Gromacs*. Root Mean Square Deviation (RMSD) and Root Mean Square Fluctuation (RMSF) were calculated using *VMD* 27. Visual representations of the simulated structures were prepared using *PyMOL* 23.

## Results

### Clinical characteristics and phenotypes

**Figure 1:**
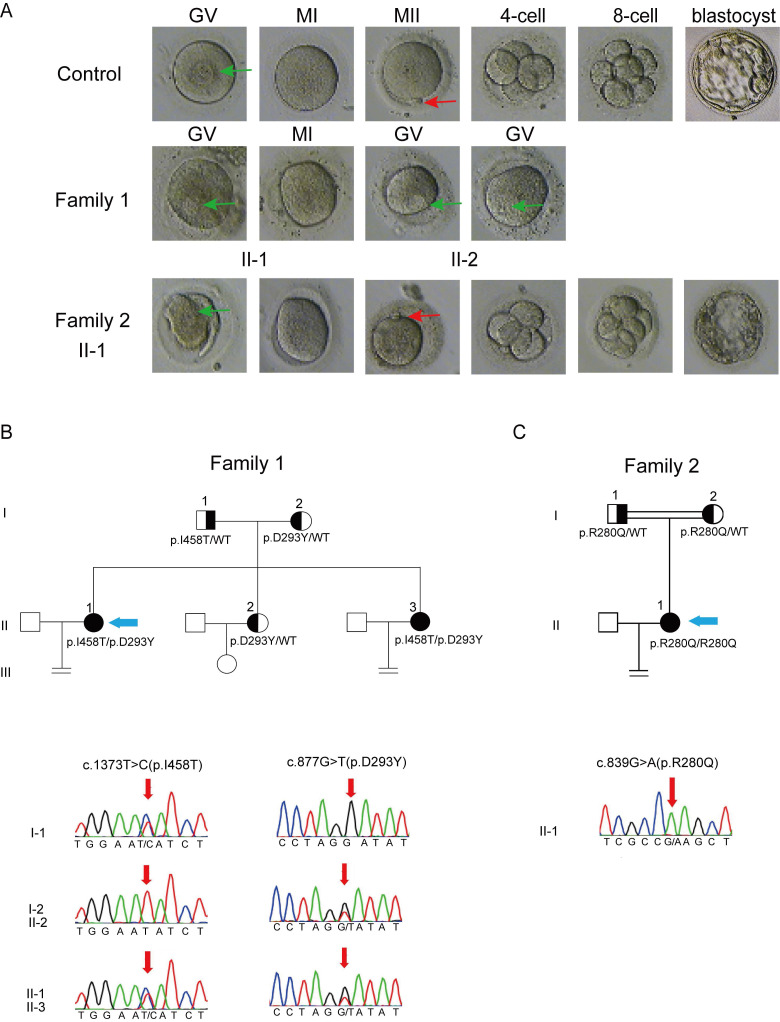
Phenotype of oocytes retrieved from the affected patients and pedigree analysis of the two families harboring variants in *PATL*2. (A), Morphology of oocytes from a control and the affected subjects; The first polar body and the germinal vesicle are indicated by red and green arrows, respectively. (B and C), Pedigrees of family 1 (B) and 2 (C) with variant identification in *PATL*2 confirmed by sanger sequencing. The probands are indicated by blue arrows.

In family 1 that has no history of consanguinity, patients II-1 and II-2, 33 and 26 years old, are sisters having been diagnosed with primary infertility for 4 and 3 years, respectively. Both patients exhibited regular menstrual cycles and normal sex hormone concentrations (Table 1). Patient II-1 experienced 2 IVF attempts, with a total of 17 oocytes retrieved: 14 arrested at the GV stage, 2 arrested at the MI stage and 1 being morphologically abnormal; patient II-2 underwent 1 IVF cycle, with a total of 29 oocytes retrieved: 24 arrested at the GV stage and the remaining oocytes displaying morphologic abnormality. In summary, all retrieved oocytes from both patients were either immature or morphologically abnormal (Fig. 1A), which unexceptionally led to complete fertilization failures.

Patient II-1 from a consanguineous family (family 2), 26 years old, was diagnosed with primary infertility after 5 years of attempting to conceive. She had regular menstrual cycles and normal sex hormone concentrations (Table 1), but showed different clinical manifestations from the patients in family 1. In her first IVF cycle carried out at Chongqing Health Center for Women and Children, a total of 23 oocytes were retrieved, with a high proportion of MII oocytes (18). Seven of these oocytes were successfully fertilized, yielding 2 cleavage-stage embryos (Fig. 1A), but there was no pregnancy after implantation. In her second IVF cycle carried out at the Reproductive Medical Center, Daping Hospital, 14 out of 21 retrieved oocytes were at the MII stage. Most of them were successfully fertilized, as evidenced by the observation of two pronuclei. However, only 2 cleavage-stage embryos and 2 blastocyst-stage embryos were obtained (Fig. 1A). Similarly, pregnancy failed again after embryo transfer. Detailed information regarding the clinical phenotypes of all patients is given in Table 2.

### Identification of novel variants in *PATL*2

To identify potential genetic causes of primary infertility in the patients described above, we performed whole exome sequencing (WES) on their DNA samples. The two patients in family 1 were found to harbor compound heterozygous variants of *PATL*2 (NM_001145112.1), consisting of a novel missense variant, c.1373T>C (p.I458T), and a previously reported missense variant, c.877G>T (p.D293Y), both of which were verified by Sanger sequencing (Fig. 1B). Variants in other 21 genes having been reported to be associated with oocyte/zygote/embryo maturation arrest, such as *TUBB*8, *NLRP*2*/NLRP*5, *ZP*1*/ZP*3, and *PANX*1 (Table S1), were not found. Sequencing of the *PATL*2 gene from their parents confirmed that the p.I458T and p.D293Y variants were descended from the father and mother, respectively. The patients in this family have another sister who is fertile and carries a monoallelic variant of p.D293Y inherited from the mother (Fig.1B). The allele frequencies of both p.I458T and p.D293Y variants are currently unknown, as they are not listed in the 1000 Genomes, ExAC Browser, GnomAD Browser, or referenced in the clinical variant database (ClinVar, www.ncbi.nlm.nih.gov/clinvar/). The p.D293Y variant is documented in human gene mutation database (HGMD, www.hgmd.cf.ac.uk/ac/) with accession number CM197370. Both variants are predicted with high probability to be clinical deleterious on the websites of SIFT, Polyphen-2, and Mutation Taster, although both of them are currently classified as variants of uncertain significance according to the ACMG guidelines (Table 3).

In Family 2, which has a history of consanguinity, the patient was found to carry homozygous variants of c.839G>A (p.R280Q), in the *PATL*2 gene. This missense variant has been reported but was identified from a patient harboring compound heterozygous variants 12. Therefore, the homozygosity of this variant identified in this study is unprecedented. The allele frequency of this variant site ranges from 0.5‰ to 2.0‰ and its genetic load is predicted to be very likely damaging by SIFT, Polyphen-2, and Mutation Taster, though classified as a variant of uncertain significance according to the ACMG guidelines (Table 3).

### Variant sites in the protein sequence and structure of PATL2

**Figure 2:**
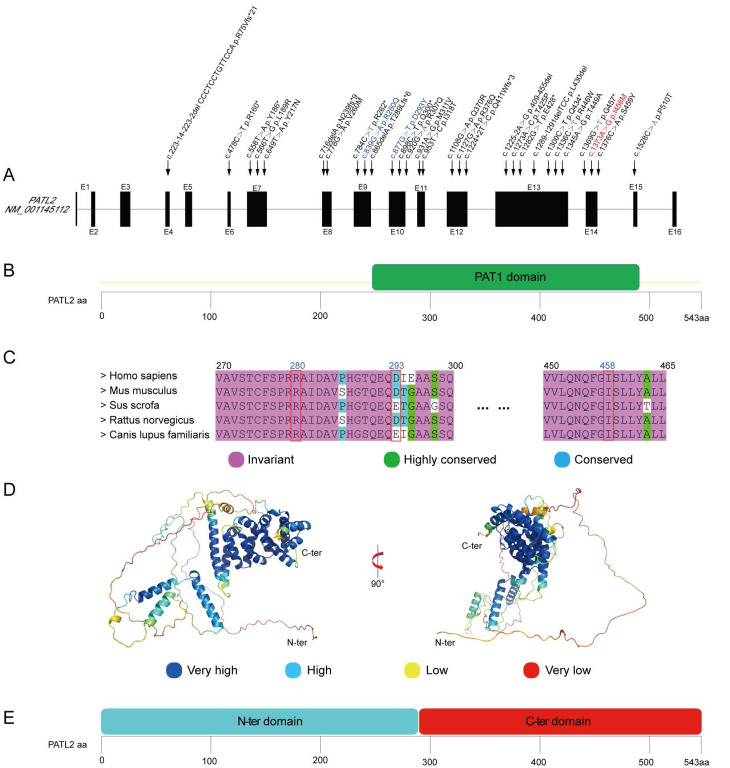
Analysis of the identified missense variants occurring in the PATL2 protein. (A), Positions of known variants in the genomic structure of PATL2. The novel variant p.I458T, the two recurrent variants p.R280Q and p.D293Y, and all other reported variants were labelled in red, blue, and black, respectively. (B), Domain organization of PATL2 from the UniProt Database (www.uniprot.org). (C), Sequence alignment of the PATL2 among human, mouse, swine, rabbit, and dog with a color index according to amino acid conservation. The three variant sites identified in this study are indicated by red boxes. (D), A predicted 3D model downloaded from AlphaFold Protein Structure Database (alphafold.com). Colors represent the predictive confidence for each residue, with the color cards corresponding to different confidence scales given bellowed the structure representation. (E), Module organization schemed from structure prediction.

The three identified variants, p.R280Q, p.D293Y, and p.I458T, occur in exons 9, 10, and 14 in the *PATL*2 gene, respectively. These exons have previously been reported to harbor multiple variant sites (Fig. 2A). Notably, all these three variants are located within the PAT1 domain (residues 252-491), which is defined as a homologue to the Pfam domain PF09770 (Fig. 2B). Sequence alignment shows that the amino acids at positions 280 and 458 are invariant in PATL2 orthologues from various species, highlighting their critical functional and/or structural roles. The residue at position 293 is occupied by an aspartic acid or a glutamic acid, both of which are negatively charged residues and thus possess very similar chemical properties (Fig. 2C). In another word, all the three variant sites identified in this study correspond to highly conserved positions, simply like other reported sites in *PATL*2 where missense variants were identified 12, 17, 28.

Each of the three variants identified in this study introduces an amino acid with different chemical property from the original one. In details, R280Q changes a positively charged Arg residue to a neutral hydrophilic Gln residue; D293Y substitutes a negatively charged Asp residue with an aromatic Tyr residue; I458T replaces a hydrophobic Ile residue with a polar Thr residue. Theoretically, all these substitutions may destabilize local structure of the PATL2 protein by changing the chemical features of local regions. To assess this possibility, we firstly predicted the free energy change (ΔΔG) from the wild-type sequence to each variant using three algorithms including mCSM 29, SDM 30, and DUET 21 on a webserver. The predicted ΔΔG values for the three variants, however, did not arrive at convergent outputs (Table S2). While I458T was clearly predicted to destabilize the protein structure (ΔΔG < 0), both D293Y and R280Q showed varying ΔΔG values among the three algorithms. This inconsistency suggests that the structural impacts driven by these two variants are not straightforward and thus require further investigation.

To gain a more plausible understanding of structural impact resulting from the identified variants on the PATL2 protein, we downloaded a full-length predicted PATL2 structure from AlphaFold Protein Structure Database (AlphaFoldDB) 22, as *AlphaFold* is thought to be one of the most robust AI-based algorithms for protein structure prediction 24. The overall model of intact PATL2 is apparently composed of two independent structural domains (Fig. 2D). The N-terminal domain (residues 1-289) appears to be highly mobile, comprising only three α-helices separated by long disordered loops. Noteworthily, these disordered regions are marked with low predictive confidence by *AlphaFold*. In sharp contrast, the C-terminal part (residues 290-543) forms a compact all-α-helix domain with a very similar fold to PAT1 C-terminal domain (PDB entry 2XEQ) 31, and is predicted with high degree of confidence. Interestingly, the boundaries of the C-terminal domain predicted based on structural similarity do not strictly align with those defined by sequence homology (Fig. 2E and 2C). This discrepancy definitely argues for the importance of taking both protein sequence and structure into consideration when assessing possible impacts on protein function and structural stability arising from the clinically identified variants.

### Molecular dynamics simulations of wild-type and mutant PATL2

**Figure 3:**
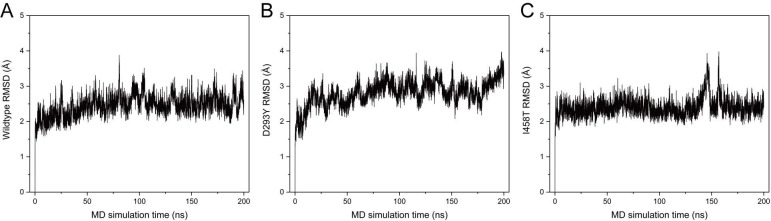
The Root Mean Square Deviation (RMSD) distributions of the C-terminal PAT1 domain (residues 290-543) of wild-type PATL2 (A), the D293Y mutant (B), and the I458T mutant (C) throughout 200 ns of MD simulation.

**Figure 4:**
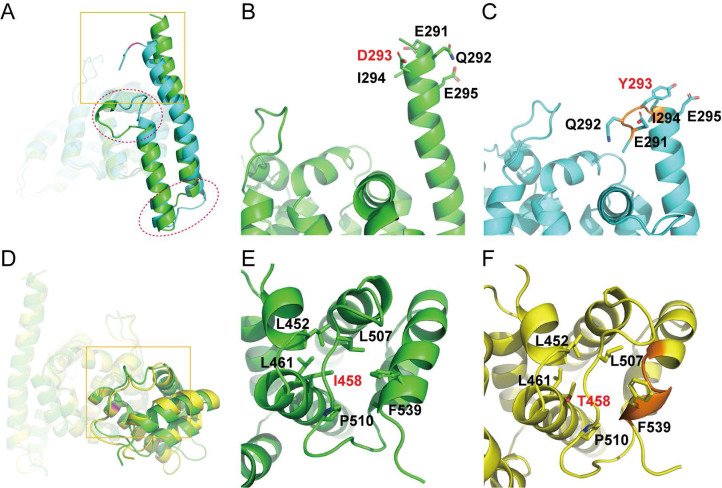
Representative structures of the C-terminal domain in wild type PATL2 and the D293Y and I458T mutants retrieved from the MD simulation trajectories. (A and D), overlaid structures of the WT protein onto the D293Y mutant (A) or the I458T mutant (D). Secondary-structural differences are denoted by red circles, while the conformational discrepancies around the mutation site are indicated by yellow boxes. (B and C), Close view of the local conformation encircled by the yellow box in panel (A). (E and F), Close view of the local conformation encircled by the yellow box in panel (D). In all panels, WT PATL2, the D293Y mutant, and the I458T mutant are colored in green, cyan and yellow, respectively. In panels (B, C, E, and F), the mutation sites and adjacent amino acids are shown in sticks. Local collapses of secondary structures were indicated in orange color (C and F).

To obtain more reliable prediction of potential impacts on the structural integrity of PATL2 upon the identified variants in this study, we performed MD simulations using the C-terminal domain from the *AlphaFold*-predicted model as a starting structure. The N-terminal domain was not included in the simulations because of its much lower predictive confidence (Fig. 2D). Mutants of D293Y and I458T were built by *in silico* residue replacement. The wild-type (WT) protein and the two mutants all showed stable RMSD distributions throughout 200 ns simulations (Fig. 3), indicating stereochemical correctness of the predicted structure and acceptable simulation trajectories. Compared with WT PATL2, however, the mutants displayed apparent changes in local secondary structures (Fig. 4).

The mutation site of D293Y occurs at the N-terminus of a long α-helix (residues 291-323) close to domain surface, which partially collapsed at the beginning of the MD simulation (~ 8 ns) and lasted to the end. Furthermore, this structural damage was propagated to another downstream α-helix (residues 331-347), and led to other local conformational changes at the connecting loop between the two helices and even the peptide following the second helix (Fig. 4A). These structural alterations could be ascribed to the introduction of a Tyr residue with bulky side chain at position 293 (Fig. 4B), which causes steric hindrance with neighboring amino acids and hence abolishes one turn of at the N-terminus of the long α-helix (Fig. 4C).

In contrast, the mutation site of I458T is positioned at a hydrophobic core buried inside the C-terminal domain of PATL2 (Fig. 4D) and surrounded by hydrophobic residues including L452, L461, L507, P510, and F539 (Fig. 4E). Apparently, the replacement with a hydrophilic residue at this position would definitely ruin the hydrophobic core, and consequently, the C-terminus of a vicinal α-helix (residues 527-541) was deformed into a short 3_10_-helix in most time of the simulation (62-161 ns) (Fig. 4F).

These observations from our MD simulations clearly indicate that compared to wild-type PATL2, both D293Y and I458T mutants showed significantly destabilized local structural arrangements around the mutation sites and may induce partial collapse of the vicinal secondary structures. We thus reason that these mutations would likely impede the proper folding of PATL2 after protein synthesis, which theoretically results in rapid degradation of the accumulated misfolded protein by specific proteases within the cell, leading to total loss of the PATL2's function. This speculation, based our simulation data, seems to provide a plausible explanation for the previously observed sharply decrease of protein amount but comparable mRNA level upon some reported PATL2 variants 12, 16, 20, 28.

## Discussion

Along with the prevalent applications of assisted reproductive techniques in clinic settings, previously unobserved infertile phenotypes have been revealed. Since the first identification of *PATL*2 variants as a genetic cause for female infertility in 2017 12, 13, the identified variants have been associated with a variety of clinical phenotypes, including immature oocyte production, unsuccessful fertilization, and arrested early embryonic development 14-17, 28. It has been hypothesized that phenotypic variability may depends on the impairing extent of the PATL2 protein, i.e. severer functional abolishment of this protein may result in disorders at an earlier stage 12. However, the detailed mechanism underlying the correlation between genetic variants and phenotypic manifestations remains undermined, largely attributed to the unknown physiological functions of PATL2.

In mammalian oocytes, a substantial amount of the maternal mRNAs is accumulated to support subsequent oocyte maturation and embryonic development 32, but up to 30% of theses mRNAs are translationally silenced by polyadenylation and stored in cytoplasmic granules until meiotic maturation 33. Multiple RNA-binding proteins may participate in the process to maintain mRNA homeostasis in oocytes through the formation of very large complexes of ribonucleoproteins (RNP) 34. PATL2 is regarded as an mRNA-binding protein playing crucial roles from oocyte maturation to embryonic development, as demonstrated by phenotyping in *Patl*2 knockout mouse models 14, 20. As a translational repressor, PATL2 may cooperate with other mRNA-binding proteins such as CPEB, MSY2, DDX6, and EIF4E in RNP 14, 20, but the precise temporal and spatial regulation of PATL2 expression, its bioactivity, and its interplay with binding partners in RNPs remain unclear.

In this study, three patients from two unrelated families who experienced recurrent IVF failures were identified to harbor biallelic missense variants in *PATL*2, yet displayed different clinical manifestations. The oocytes retrieved from the affected sisters in family 1 were all arrested at the GV stage, whereas the majority of those retrieved from the patient in family 2 were at the MII stage, with transferable embryos harvested but failed pregnancy after implantation. This phenotypic difference may stem from the differing functional impacts of the identified variants. The loss-of-function (LoF) variants D293Y and I458T likely cause severe impairments of PATL2 function by significantly reducing protein abundancy, while the R280Q variant probably does not lead to complete function loss, which could be classified as hypomorphic (Hyp) according to Mendelian principles.

The impact of these variants on oocyte maturation and early embryonic development can be further understood through their roles in specific cellular pathways. Oocyte maturation requires precise regulation of meiotic progression and cytoplasmic preparation for fertilization. Complete LoF variants, such as D293Y and I458T, disrupt these processes, leading to severe maturation arrest at the GV stage. Conversely, the residual protein activity of an Hyp variant like R280Q may partially support meiotic progression, thereby avoiding severe oocyte maturation failure. In the context of early embryonic development, a different threshold effect may exist. The Hyp variant in PATL2 may not possess sufficient protein activity to support full embryonic development, ultimately resulting in failed pregnancy. In this sense, the compound heterozygous LoF/LoF variants carried by the sisters in family 1 reach the pathogenetic threshold for GV oocyte arrest, while the homozygous Hyp/Hyp variants harbored by the individual in family 2 reach the threshold for developmental failure in a later stage after embryo cleavage. This model of gene-disease relationship underscores the importance of quantitative protein function in developmental viability and suggests that PATL2's role in oocyte/embryo biology is probably dosage-sensitive, with distinct phenotypic outcomes emerging from variant-specific functional deficits 35.

An alternative scenario is that PATL2 may have multiple biochemical activities corresponding to different domains. Specifically, the C-terminal PAT1 domain of PATL2 might be primarily responsible for binding mRNA and other cooperative proteins during the GV oocyte stage, while the N-terminal domain plays a relatively minor role at this stage. In the stage of embryonic development, however, the N-terminal domain might become more critical, potentially engaging in interactions with other PATL2 interaction partners different from those at earlier stages. Under this hypothesis, the homozygous variant p.R280Q, located in the N-terminal domain, might be tolerable during oocyte maturation but become intolerable at a later stage after embryo transfer. This scenario underscores the complexity of PATL2's role in reproduction, suggesting that different domains of this protein may have distinct functions at various stages from oocyte maturation to embryonic development.

In summary, the novel variants of *PATL*2 identified in our study further expand the genotypic and phenotypic spectra associated with this gene. The mechanism underlying the relationship between genetic and phenotypic variations warrants more in-depth studies on the structure and bioactivities of PATL2, with the hope of greatly increasing our knowledge regarding PATL2 biology, which is essential for predicting the pathogenicity of novel genetic variants and evaluating the necessity of IVF/ICSI treatments.

## Supplementary Material

Supplementary tables.

## Figures and Tables

**Table 1:** Sex hormone concentrations in the affected subjects

Patient		FSH (mIU/mL)	LH (mIU/mL)	E_2_ (pg/mL)	P (ng/mL)	PRL (ng/mL)	T (ng/mL)	AMH (ng/mL)	AFC
Family 1	II-1	10.89	3.47	31.84	0.99	36.41	0.47	1.08	6
	II-2	5.89	2.84	68.45	0.54	16.51	0.51	7.71	16
Family 2	II-1	5.81	7.3	35.99	1.13	18.83	0.46	2.34	10
Reference ranges		3.85-8.78	2.12-10.89	15.16-127.81	0.31-1.52	3.34-26.72	0-0.75	0.17-7.37	≥7

**FSH**: Follicle-Stimulating Hormone. **LH**: Luteinizing Hormone. **E_2_**: Estradiol. **P**: Progesterone. **T**: Testosterone. **PRL**: Prolactin. **AMH**: Anti-Müllerian Hormone. **AFC**: Antral Follicle Count.

**Table 2:** Clinical characteristics of the affected subjects and their retrieved oocytes

Patients		Age (Years)	Duration of Infertility (Years)	IVF/ICSI cycles	Total no. of Oocytes retrieved	GV^a^ oocytes	MI^b^ oocytes	PB1^c^ oocytes (MII^d^)	Oocytes with abnormal morphology	Degenerated oocytes	Fertilized oocytes	Embryos that could be cleaved	Embryos arrested at an early stage
Family 1	II-1	33	4	2	17	14	2	0	1	0	0	0	0
	II-2	26	3	1	29	24	0	0	5	0	0	0	0
Family 2	II-1	31	5	2	44	0	8	32	2	4	19	19	14

^a^ GV: germinal vesicle. ^b^ MI: meiosis I. ^c^ PB1: polar body 1. ^d^ MII: meiosis II.

**Table 3:** Summary of *PATL*2 variants detected in the affected subjects

Patient		Genomic position on Chr15(bp)	cDNA change	protein change	Exon position	Mutation type	Genotype	SIFT^a^	PPH2^a^	Mutation taster^a^	1KG_eas^b^	ExAC_eas^b^	gnomAD(East Asian)^b^	ACMG classification^c^
Family 1	II-1	44959394	c.1373T>C	p.I458T	14	missense	Heterozygous	0.01(T)	0.999(P)	0.999(D)	NA	NA	NA	VUS
	II-2	44961761	c.877G>T	p.D293Y	10	missense	Heterozygous	0.02(T)	0.963(P)	0.987(D)	NA	NA	NA	VUS
Family 2	II-1	44962012	c.839G>A	p.R280Q	9	missense	Homozygous	0.02(T)	1.0(P)	0.996(D)	0.0002	0.00005	0.000054	VUS

^a^ Mutation assessment by SIFT, PolyPhen-2 (PPH2), and Mutation Taster. T: tolerance, P: probably damaging, D: damage. ^b^ Frequencies of corresponding mutation in East Asian population of 1000 Genomes (1KG), ExAC Browser, and GnomAD. NA: not available. ^c^ The pathogenicity classification of the variants according to the ACMG guidelines. VUS, variant of uncertain significance.
