# Supplementary material for: Novel Genetic Variants in PATL2 Corresponding to Different Clinical Phenotypes of Female Infertility
Source: Int J Med Sci. 2025 Jun 23;22(12):3132–41. doi: 10.7150/ijms.109085 (PMC12243965; doi:10.7150/ijms.109085)
Supplement: Supplementary file 1 — Supplementary tables. [file ijmsv22p3132s1.pdf]

Novel Genetic Variants in *PATL2* Corresponding to Different Clinical Phenotypes of Female Infertility

Xiaotao Yang[1, ‡], Xiangrui Shi[1, ‡], Jing Wang[1], Jingying Guo[1], Yinhu Huang[1], Pan Tang[1], Yu Zhao[1], Yanxi Li[1], Wei Liu[1, 2, *], Qinghua Zhang[1, *]

[1]Reproductive Medicine Center, Daping Hospital, Army Medical University, Chongqing, 400042, China

[2]Institute of Immunology, Army Medical University, Chongqing, 400038, China

‡ These authors contributed equally to this work.

*** Corresponding authors:** weiliu@tmmu.edu.cn (W. Liu) and zhangqh@tmmu.edu.cn (Q. Zhang)

Table S1 List of genes examined

Gene	Phenotype	Inheritance*
**PADI6**	Oocyte/Zygote/Embryo Maturation Arrest Type 16	AR
**CDC20**	Oocyte/Zygote/Embryo Maturation Arrest Type 14	AR
**ZFP36L2**	Oocyte/Zygote/Embryo Maturation Arrest Type 13	AR
**ASTL**	Oocyte/Zygote/Embryo Maturation Arrest Type 11	AR
**TRIP13**	Oocyte/Zygote/Embryo Maturation Arrest Type 9	AR
**ZP3**	Oocyte/Zygote/Embryo Maturation Arrest Type 3	AD
**KPNA7**	Oocyte/Zygote/Embryo Maturation Arrest Type 17	AR
**WEE2**	Oocyte/Zygote/Embryo Maturation Arrest Type 5	AR
**MOS**	Oocyte/Zygote/Embryo Maturation Arrest Type 20	AR
**FBXO43**	Oocyte/Zygote/Embryo Maturation Arrest Type 12	AR
**TUBB8**	Oocyte/Zygote/Embryo Maturation Arrest Type 2	AD/AR
**ZP1**	Oocyte/Zygote/Embryo Maturation Arrest Type 1	AR
**PANX1**	Oocyte/Zygote/Embryo Maturation Arrest Type 7	AD
**BTG4**	Oocyte/Zygote/Embryo Maturation Arrest Type 8	AR
**CHEK1**	Oocyte/Zygote/Embryo Maturation Arrest Type 21	AD
**PATL2**	Oocyte/Zygote/Embryo Maturation Arrest Type 4	AR
**REC114**	Oocyte/Zygote/Embryo Maturation Arrest Type 10	AR
**ZP2**	Oocyte/Zygote/Embryo Maturation Arrest Type 6	AR
**TLE6**	Oocyte/Zygote/Embryo Maturation Arrest Type 15	AR
**NLRP2**	Oocyte/Zygote/Embryo Maturation Arrest Type 18	AR
**NLRP5**	Oocyte/Zygote/Embryo Maturation Arrest Type 19	AR
**PABPC1L**	Oocyte/Zygote/Embryo Maturation Arrest Type 22	AR

* AD: autosomal dominant, AR: autosomal recessive.

Table S2 PATL2 variants stability predicted by online server.

ΔΔG (k_{cal}/mol)	R280Q	D293Y	I458T
mCSM	-0.003	0.028	-3.004
SDM	-0.65	-0.13	-2.69
DUET	0.02	0.02	-3.236
